# Quality of life in childhood advanced cancer: from conceptualization to assessment with the Advance QoL tool

**DOI:** 10.1186/s12904-022-01025-z

**Published:** 2022-08-01

**Authors:** Josianne Avoine-Blondin, Émilie Dumont, Marc-Antoine Marquis, Michel Duval, Serge Sultan

**Affiliations:** 1 Charles-Bruneau Cancer Care Centre, Sainte-Justine UHC, Research Centre, 3175, Chemin de la Côte-Sainte-Catherine, Montréal, QC H3T 1C5 Canada; 2 Faculty of Postgraduate Studies, Université de Montreal, Montreal, QC Canada; 3 Department of Psychology, UQÀM, Montréal, QC Canada; 4 Department of Pediatrics, Université de Montreal, Montreal, QC Canada; 5 Department of Psychology, Université de Montreal, Montreal, QC Canada

**Keywords:** Palliative care, Advanced cancer, Pediatrics, Quality of life, Mixed-method

## Abstract

**Background:**

Advanced childhood cancer, a condition with no available cancer-focused treatment options, greatly impacts Quality of Life (QoL). We need appropriate assessment strategies to select adapted treatment targets, improve care and optimize communication. Our first goal was to identify the domains of patients’ QoL by combining for the first time the perspectives of patients and parents with previously collected reports in professionals. Our second goal was to develop a simple QoL assessment tool and optimize its format and content for use in the childhood advanced cancer population.

**Methods:**

To identify QoL domains, we conducted qualitative interviews with 7 young patients (4 girls, 3 boys, aged 13 ± 4 yrs) and 9 parents (7 mothers, 2 fathers) from our treatment centre. We used inductive thematic content analysis to code and categorize respondents’ viewpoints. The first version of the tool (Advance QoL) was then drafted, and structured feedback was collected through interviews and a survey with 15 experts. We computed content validity indices.

**Results:**

Apart from the physical, psychological, and social domains, participants insisted on four original themes: autonomy, pleasure, the pursuit of achievement, and the sense of feeling heard. This was in line with the categories found in a preliminary study involving professionals (PMID: 28137343). Experts evaluated the tool as clear, relevant, acceptable, and usable. They formulated recommendations on instructions, timeframe, and item formulations, which we implemented in the refined version.

**Conclusions:**

Advance QoL is an innovative tool targeting key life domains in childhood advanced cancer. It is focused on preserved abilities and targets of care. The refined version is appropriate for adult respondents within families and professionals. Future studies will develop versions for young ages to collect the experience of patients themselves. This will open on future reliability, validity, sensitivity, and implementation studies.

**Supplementary Information:**

The online version contains supplementary material available at 10.1186/s12904-022-01025-z.

## Introduction

Cancer is a life-threatening disease which imposes major challenges for children and their entourage. Along the trajectory, children experience substantial suffering and uncertainty as a result of the disease and treatments [1]. For some children, existing cancer-focused treatments may reveal ineffective, and the illness becomes an “advanced cancer”, a critical state experienced by tens of thousand of young people in North America [2]. In such context of uncertainty, children and their family see their daily lives disrupted and their overall quality of life (QoL) widely affected [2–5].

Monitoring QoL is useful to facilitate treatment and adjust support provided to families [2, 5–7]. A variety of tools have been used to understand the impact of the disease on patients’ QoL and guide clinical decision-making [2, 8]. However, it has been suggested that existing tools are often not appropriate for children with advanced cancer. Researchers have consistently recommended adapting instruments or developing new ones for this specific population [8–10]. A systematic review revealed validity issues with existing assessment instruments [9]. The vast majority of reviewed measures did not use an appropriate time lapse for the recall period (e.g. 1 month) and did not cover adequately the experience of children with a life-threatening disease [8, 9]. Healthcare professionals from a pediatric hematology-oncology department also reported lacking an appropriate and feasible tool to estimate the QoL of patients receiving palliative care [11].

A pool of items was recently chosen for this population to develop a scale [12, 13]. The items were initially selected from the literature and cover physical comfort, psychological well-being, social interactions, resilience, and quality of care [12, 13]. It is unclear however if this list of domains is comprehensive and if it reflects the experience of end-users. To confirm this, inductive approaches are essential, as they help identify themes that matter most to children and their families. Recently, a fully inductive qualitative design investigated the views of pediatric hematology-oncology professionals on how to define QoL in the context of childhood cancers who received palliative care [14]. The authors found that professionals referred to seven life domains: physical comfort, alleviation of psychological suffering, fun and the present moment, sense of control, feeling that life goes on, feeling valued and appreciated, and meaningful social relationships. To date, we do not know whether patients and families would endorse the same definition.

Confirming these target domains with families is an essential preliminary step prior to developing an assessment tool [8, 15, 16]. Knowing how to conceive of QoL in this context would make it possible to choose indicators likely to reflect key characteristics of QoL domains. For instance, one might choose to inquire whether the patient enjoyed her favorite meal to grasp the domain “experiencing day-to-day pleasure”. It is also essential to select an appropriate timeframe as there are strong arguments suggesting that 1-month or 1-week timeframes used in existing instruments prevents from capturing the variability of some important themes in the context of a life-threatening disease [9, 14].

In sum, the literature shows that domains that matter most to families have not yet been formulated by children and parents, and that there is no tool appropriate to the context of childhood advanced cancer. The present report describes part of the process aiming at developing a pertinent and feasible assessment of QoL aiming at supporting clinical judgment and help communicate on QoL. We had two objectives: [1] We wished to identify the domains of QoL from the points of view of patients and parents. This first objective aims at complementing data previously collected with healthcare professionals and synthesizing the views of children, parents, and professionals. Based on the results, we elaborated a simple tool able to grasp the key life domains of this population [2]. We wished to optimize this preliminary version by testing its content and format with experts and collected a structured feedback.

### Context of the study

The study was conducted at the Centre Hospitalier Universitaire (CHU) Sainte-Justine, a pediatric hospital affiliated to the University of Montreal and located in Montreal, Canada. The study was made possible by the collaborative work between the Centre de Psycho-Oncology (CPO), the Pediatric Palliative Care Unit and the Hemato-Oncology Department of CHU Sainte-Justine. Here is a brief description of the authors and researchers involved in this study to provide an idea of their contributions and gauge the influence of their background on the study process.

JAB is a child psychologist and postdoctoral fellow at CPO. Her first experience in pediatric oncology was acquired through volunteer activities with the Hemato-Oncology Department at the CHU Sainte-Justine and other organizations in the field. She completed a clinical psychology doctorate (D.Psy) in the field of pediatric palliative care and joined the Quebec Research Network on Palliative Care (Réseau Québécois de Recherche en Soins Palliatifs, (RQSPAL) as president of the Student committee and member of the scientific committee. She conducted the study under the supervision of SS and MD. Throughout the study, she favoured a humanistic and constructivist approach. This way, she offered a space of trust for children and parents to feel free to express their own reality. SS is the research unit director of the CPO. Over the last decade, he has developed an expertise in pediatric psycho-oncology. His research activities aim to promote QoL for sick children and their families. Guided by an evidence-based approach, he helped structured the vision of the study. MD is a hematologist/oncologist with a long experience in clinical ethics (research and practice). As head of the Hematology-Oncology Department of CHU Sainte-Justine, he helped to connect with the member of the clinical department and provided a clinical vision throughout the study. MAM is a pediatrician and director of the Palliative Care Unit. Through his experience in the field of pediatric palliative care and his specialization in pain management, he provided a specific clinical view. ED is a master student in pediatric psychology. As an associate of the CPO she helped with transcription and analysis of the qualitative data.

This study is a continuation of our previous studies conducted with healthcare professionals at CHU Sainte-Justine [11, 14]. Beyond the purpose of contributing to the advancement of knowledge on the definition of the QoL in the field of PPC, we hoped the results could improve awareness about the domains of QoL among healthcare professionals in our institution. The ultimate goal of the tool from this study is to collect information likely to be shared within the team and with the family, and thus improve communication in the context of advanced cancer.

## Methods

The study is based on a sequential mixed method design. We used an inductive and descriptive qualitative method to meet the first objective, while we applied qualitative-quantitative survey methods to meet the second objective. The study received ethical approval from the Sainte-Justine UHC Research Ethics Committee (2018–1871). Written informed consent and assent was obtained from all the participants and their legal guardians. We used the Consolidated Criteria for Reporting Qualitative Research (COREQ) checklist to help tutor the reporting of the qualitative study (Supplementary material).

### Identification and refinement of QoL target domains

#### Participants

Participants were pediatric patients with advanced cancer and parents of such patients who were receiving care at the hematology-oncology department at Sainte-Justine UHC (Montréal, Québec, Canada). We defined advanced cancer as a refractory or relapsed illness with an unfavourable prognosis and for which no existing standard cancer-focused treatment option existed. To be included in the study, patients had to be aged between 6 and 21 years old, have advanced cancer as evaluated by one oncologist of the medical team, be capable of communicating verbally, and speak French. Parents’ inclusion criteria were to have a child with such advanced condition at the time of study and to speak French. No other inclusion or exclusion criteria were used. To determine the number of participants, we used data saturation, a methodological criterion that informs the researcher when to stop collecting data. Data is deemed saturated when new data do not provide sufficiently new information to better understand the phenomenon under study and to justify an increase of material [17]. Following this principle, we stopped recruitment after two protocols did not bring new codes for each group independently [18, 19].

#### Procedure

We used a convenience sampling method to recruit the participants. Particular attention was paid to the inclusion of children (or parents with children) of various ages, diagnoses and cultures. This diversity aimed to generate as many themes as possible in order to ensure the credibility and comprehensiveness of the data and optimize transferability [20, 21]. Children and parents who met the inclusion criteria were identified by the primary nurses and oncologists, or by nurses from the palliative care team. There was no pre-existing relationship between participants and authors directly involved in data collection, including the first author (JAB). Considering the sensitive nature of the subject of the study and to avoid inconveniencing families by introducing a new stakeholder, potential participants were informed by their primary nurses of the opportunity to participate in a QoL study. In case of interest, the primary nurse would refer them to the research team for an in-person encounter providing full information and collecting a signed consent (and assent if appropriate). Then, the principal investigator (JAB) briefly described her position and explained them the study while informing them of the confidentiality of their data, their freedom to ask any questions and to stop participating at any time without being asked for any justification and without this having an impact on their relationship with the care team. In addition, considering that interviews could arouse unpleasant emotions or provoke painful thoughts, we made sure that the person conducting the interviews had the necessary skills to accompany them with kindness during the interviews. Additionally, a follow-up phone call was made 2 weeks after the interviews and a referral to the hospital’s clinical service could be made if necessary.

We collected data through individual semi-structured interviews lasting 30–45 minutes (Aug-Dec 2018). The interviews were conducted at a time convenient to the participants and took place in our hospital, either in the research group’s consultation office or in the children’s hospital room when these were unable to move. In case of interruption by a healthcare professional, we would put the interview on hold to ensure confidentiality. Although the study did not aim to systematically study family dyads, we chose not to exclude the possibility of meeting participants from the same family, given the limited target population. So, when there was a parent and child participant from the same family, the interview was scheduled one after the other independently to avoid contamination biases. We also made it clear from the start that the purpose of the study was not to compare responses from the same families and that all material was kept confidential. Furthermore, to collect the views of children and parents based on their own current experience, and avoid inducing their perception by our a priori knowledge, the interview began with open-ended questions enquiring inductively about patients’ QoL. The questions were adapted from Hinds, Gattuso [22] and are similar to those used by Avoine-Blondin et al. with professionals [11] (Supplementary material A). As a second step, the 38 themes of all 7 domains previously identified in professionals were presented to the participants [14]. To make this presentation easy and friendly, we presented these themes on cards. Upon presenting each card, we asked participants to tell whether the theme was important to define “a good day” and to explain why. Then we asked participants to critically evaluate the pertinence and clarity of the 7-domain description previously published [14]. This two-step interview permitted to triangulate data to complement, enrich and reformulate the individual themes as well as the grouping of themes into domains. At the end of the interview, we administered a short sociodemographic questionnaire (including age, gender, family life, cancer diagnosis). The interview was conducted by the first author (JAB), recorded, and subsequently transcribed by a graduate student within our team (ED).

#### Analysis

We selected a sequential thematic analysis to produce a thematic tree of QoL domains for groups of patients and parents independently [20, 21]. The analysis was carried out with the software NVivo v11.

Firstly, we used a continual thematization process to analyze the discourse of patients and parents independently. For each group, we randomly chose 4 transcripts and double-coded them to extract codes reflecting aspects of QoL (coders: JAB, ED). This step consists of an inductive bottom-up process, permitting to create comprehensive thematic clusters with high levels of inference and ensure better credibility of the results. As we progressed, we systematically created a list of codes in a separate document for each group (patients and parents). Through discussions, coders proceeded with code comparison and grouping. At the end of this step involving 4 transcripts, we obtained an analysis sheet with themes and categories to be applied to each remaining transcript. Thematic clusters gradually took shape and thematic trees were then constructed, reflecting the unique perspective of each group, patients and parents.

As a final step, we triangulated data of the thematic trees of patients and parents from the present study with that of healthcare professionals previously published [11, 14]. We derived a general thematic tree reflecting the perspective of the three groups. At this point, we cautiously considered the comments of children and parents regarding the description of each domain to integrate their views when describing domains. Once the domains of QoL were finally formulated, we designed the preliminary version of the Advance QoL tool.

### Content and format of the tool

In this second step, we used a mixed method design to collect feedback from experts to optimize the content and format of the tool.

#### Experts

We selected experts based on their professional experience with patients with advanced cancer from the hematology-oncology department or the palliative care team in our hospital (convenience sample). They had accompanied at least one child (0–18 years old) with advanced cancer treated with palliative care and be able to speak French. We took care to gather feedback from experts from a variety of professions given the multidisciplinary nature of the tool. We also included a patient partner as an expert. All the experts approached (*n* = 15) accepted to review the preliminary version of the tool developed in the first objective: 3 physicians, 5 nurses, 4 psychosocial professionals, 1 occupational therapist, 1 care coordinator and 1 patient partner. To reflect the variety of professions and potential end-users of the tool, we included a higher number of experts than is usually recommended. Notably, five of the 15 experts had participated in the previous qualitative inquiry [14].

#### Procedure

Using a convenience sampling method, we contacted experts through professional email or in person (Aug-Sept 2019). Once they agreed to review the preliminary version of the tool, we scheduled an appointment for an in-person interview of about 1 h. This consisted in an open-ended structured interview and a quick quantitative evaluation questionnaire aiming at collecting their appreciation of the tool. The interview was conducted by the first author (JAB).

To identify issues and collect suggestions, we asked experts to remember a situation when they had cared for a child with advanced cancer. We asked them to simultaneously complete and comment on the tool’s preliminary version. This is consistent with the think-aloud method designed to test usability of instruments [23, 24]. We then asked them to critically comment on the understandability of the instrument, its perceived utility, and the content of QoL domains. As a second step, each expert was invited to complete a brief ad hoc survey to evaluate clarity (8 items), relevance (9 items), format adequacy (1 item), and usability (1 item) (Supplementary material B). The clarity of the instruction and domain description was rated on a two-point scale (unclear vs clear) and other items were rated on a 1–5 disagree-agree scale. Items targeted the core aspects of the tool (e.g. instructions, timeframe, domain formulation, etc.). Free space was available for remarks next to each item.

#### Analysis

Data were composed of field notes, responses, and comments of experts. We first used descriptive statistics and computed the Content Validity Index (CVI) to synthesize ratings on the quantitative questionnaire [25, 26]. The CVI is the proportion of positive ratings within the pool of 15 judges. For 5-point scales we considered the neutral point as a negative judgment. Given the number of experts in our study, a proportion of CVI ≥ 0.78 was a recommended threshold to consider that experts evaluated the item as adequate. Lower CVI values indicated inadequacy and would prompt corrective actions on the item [25, 26]. Thus, we conducted a continual thematic analysis of the qualitative data [20, 21] and the final themes which represented the general suggesting views of experts on the tool led to refinements on the preliminary version considering lower CVI values. These changes led to the last version of Advance QoL. Importantly, the field notes do not allow to present meaningful quotes in the results.

#### Data availability statement

Data sharing is not possible for this study for ethical reasons as individual privacy could be compromised.

## Results

### Identification of QoL domains

A total of 20 potential participants were contacted (10 children and 10 parents). The final sample consists of 7 patients (4 girls, 3 boys, aged 13 ± 4 yrs) and 10 parents (8 mothers, 2 fathers). The overall participation rate was 85% (17/20, but one mother interview could not be transcribed due to equipment failure). Patients had been mainly treated for refractory leukemia and sarcoma.

Following the qualitative analysis of responses by parents and patients, we found 16 themes describing how parents spontaneously conceive of QoL (Table 1). Eleven of these themes were shared by patients and no additional theme was spontaneously reported by this group. Afterwards, the part of the interview using cards permitted participants to talk about aspects they would not have mentioned spontaneously despite them bearing importance (e.g. Feeling heard). By considering theses new themes with the 16 previously identified and by combining them with those of professionals already collected, we established a model of QoL based on seven domains: physical, psychological, social, feeling heard, autonomy/independence, pursuit of achievements, and pleasure. For brevity, we offer below an overview of the original findings.

**Table 1:** Quality of Life themes spontaneously identified in verbal material of 7 patients and 9 parents confronted with pediatric advanced cancer

QoL domains	QoL themes	Patients	Parents
Physical	Pain management	●	●
	Physical symptoms related to management of the disease	●	●
	Energy level	●	●
	Satisfaction of basic needs	●	●
Psychological	Mood management	●	●
	Stress management	–	●
Social	Maintaining contact with friends and family	●	●
	Relationship with caregivers	●	●
Autonomy/independence	Independence	–	●
	Physical autonomy (e.g. walking alone)	●	●
Pleasure	Do an activity that the child loves	●	●
	Having pleasure eating	●	●
Achievements	Going on with activities they were doing before the illness or maintaining activities similar to other children of the same age	–	●
	Pursuit of achievement	–	●
	Making a dream come true	–	●
	Going to school	●	●

As expected, both patients and parents perceived physical, psychological and relational well-being as core aspects of QoL. Pain management was considered a central aspect (physical well-being), but the most salient domain in their responses was social connectedness (social domain).

**F2**: “Being surrounded by family and friends. I think that is the most important thing for him.”

**I :** “What is the most important thing for you to have the best possible day? **P6:** Family”

The possibility to experience moments of pleasure was also evoked by participants from the very start of the interview. For patients and parents, pleasure could be experienced through simple activities.

**P3**: “For me a good day is to have fun, not to think about illness. [ … ] I like to go shopping, uh … going to the movies. It’s less common but I mean like … going out … playing with my dog. [ … ] Sometimes, I watch hockey because it’s my favorite sport. And uh … I play a little bit of video games. It is very important to have fun, and just laughing always feels good, because … well when you usually laugh, you don’t think about something that makes you unhappy, you think about something that makes you happy … ”

Parents spontaneously insisted on how important it is for their child to be able to go on with activities they used to do before being ill, and to continue to do things as other children do, as long as these are adapted to their condition.

**M5**: “It’s important for him to play basketball. Sometimes dad goes with him just to do dribble, so they can do dribbles, but because of his collar, sometimes it’s harder [ … ] If he didn't have the collar, if he didn't have restrictions, he would still play [ … ] Because, the fact that he can’t participate affects him. It affects him a lot, that’s why we try to fill that void by accompanying him to the games [ … ] When he goes to a game, for him … , it allows him to stay in touch with the sport he loves … ”

Although not spontaneously, the patients confirmed this topic when they were presented the cards To do an activity like before the disease and To do an activity like kids of your age. A majority endorsed them, suggesting these activities contribute to a sense of normalcy. It also apparently distracts their attention from the illness.

**I**: “ Do an activity like you did before your illness » **P4:** It’s very important for me … It makes me think that I’m a little bit back to the way I was before and that the disease hasn’t affected me that much … I still think it’s important to be able to do things the way they were before because it makes me feel better … I find little activities, even if it’s minimal, I can do the same as before, I’m like … very happy.”

Children repeatedly mentioned their desire to keep up with school. They wished to maintain contact with friends and wanted to continue learning new material. Continuing school, even at the hospital or at home and as little as once a week, helped them feel they were fulfilling their potential, and again supported a sense of normalcy and connectedness.

**P5**: “Going to school is important to me, because I'm a very academic person and I like it … and school, it's the only part of my life that is normal [...] Even if you're sick, you know that you might not make it through the year, you might have to repeat a year, but you're going to have made the effort, you'll grow up anyway.”

Both groups insisted upon the need to maintain a certain degree of autonomy. However, when being presented the cards “having a sense of control” which represented this need from the perspective of professionals, participants were confused about its meaning and asked for details. Referring to autonomy was far clearer for respondents.

**M3**: “Make decisions on your own. Yes, yes, yes, she has to make decisions, if only... which pair of shoes or... We also agreed, because I put [her] fentanyl patches … she knows it's to remove the pain, the sores, but she must see them. It's okay for me to put them on, but she has to have access … in her eyeline, she has to see them, because her nurse said at one point that we can put them on her back, but she was like, “no, no, no, no, I have to see them.” So, she handles it ...”

Respondents also confirmed the importance for patients of feeling heard and recognized by others as individuals beyond the disease.

**P3**: “When someone asks you “Are you ok?” “Do you have the right medication?” I feel reassured, because … like … they really want you to feel good, and you know … you know you’re saying something and they’re going to do everything they can to help you feel better.”

**M8**: “To be listened to, I think it is already a basis to feel appreciated, loved. [ … ] If someone listens to us, even if she doesn’t bring or say anything major, but the … uh … That’s what is going to have an impact on them, the fact that they feel listened to and understood.”

To summarize, we found that the verbal material on QoL was consistent across groups of patients and parents and that themes resembled those previously reported by professionals in the context of advanced cancer. The themes could be synthesized in 7 life domains. Based on this finding, we formulated a general model of QoL applicable to pediatric advanced cancer (Table 2). As much as possible, we used the wording of patients and parents from the present study to describe domains and their corresponding themes. For instance, we replaced “sense of control” by “autonomy” to improve understandability and avoid professional jargon.

**Table 2:** Model of Quality of Life in pediatric advanced cancer based on perspectives of patients and parents from the present study (*N* = 16) and professionals from a previous report (*N* = 20)^a^

QoL domains	Themes	Examples of verbal statements
Physical	Pain management Physical symptoms management Energy level Satisfaction of primary or basic needs	M1: “If you take away the fatigue, he could cope much better with his illness.” P1: “A day where I have the energy to do certain things.”
Psychological	Emotional distress management Cognitive symptoms management Coping with the illness	M8: “There is grief that had to be made, there are changes that were not wanted and that were imposed. So, it creates anger, dissatisfaction, uh …” P6: “Psychologically soothe, it is sure that when you have anxiety like me, it is important to feel relief.”
Social	Maintaining contact with friends and family (parents, siblings, friends, …) Positive relationships with healthcare professionals	F1: “Well, it’s seeing people … Being in contact with her sisters or her grandmothers […] Every day, we try to socialize […] So, she calls her grandmother: “Come for a walk. Come, at what time are you coming?” and then, if she can’t, she calls someone else [laughs] …” ` P4: “Keeping in touch with your friends is also important. That’s what really helped me in my treatments... because I knew that my friends were always there to support […] They often came to see me and I found that really good and it didn’t make me feel apart from them.”
Autonomy/independence	Feeling enough powerful and free to make one’s own decisions Feeling enough powerful and free to do things on his/her own Living moments of freedom and independence	M7: “Doing things by yourself, well that’s for sure important. “I’m capable now!”, like, “let me do it, I’m capable” … You know, she’s a teenager now.” P5: “I think, the more involved you are, the less powerless you feel. Because yes you don’t control your health at all, but you are able to control your care, you are able to have a voice in your care, you are able to say: “ok, I don’t want that, I would like to find a plan B” or... the more involved you are, the less powerless you are, because it’s true that you are powerless as soon as you have a diagnosis.”
Pleasure	Laughing Doing one or more activities that the child enjoys Feeling pleasure eating	M3: “[…] When she gets to play, have fun, be able to play with her brothers, share, use her colors, do … things she likes […] play with dolls, play … that’s still a good day.” P3: “For me a good day is to have fun, not to think about the disease. [...] I like to go shopping, uh... go to the movies. It’s rarer that we go to the movies, but I mean like... I go out... like play with my dog. [...] sometimes I watch hockey, because it’s my favourite sport. And uh... I play video games a little bit. It’s very important to have fun, and well just laughing always feels good, because... well when you usually laugh, you don’t think about something that’s... that makes you unhappy, you think about something that makes you happy...”
Pursuit of achievement	Going on with activities the child used to have before being ill Having similar activities as other children of the same age Feeling achievement through an activity adapted to one’s condition Making dreams or wishes come true	F2: “Right now, it’s hockey, so uh … he follows us into the arenas, he’s with me behind the bench, or sometimes he even takes to the ice when he can. So, he can return a little bit to a normal life.” P5: “You don’t have to be afraid to... to have too many dreams. We’re sick, but if you don’t dream, it’s just going to be painful right now. “
Feeling Heard	Feeling listened to and being informed of follow-ups Feeling considered as a person beyond the symptoms related to the illness	M5: “To be listened to, oh yes, yes. He wants us to be listened to by the team, it’s important to him.” P7: “Being consulted in decision-making about your care, that’s important. So I know what’s going on …”

^a^From Avoine-Blondin et al. (2017). *Palliative and Supportive Care, 15* [5], 565–574. PMID: 28137343

### Development of Advance QoL

In line with this operational definition of QoL in advanced cancer, we elaborated the preliminary version of the Advance QoL tool. Its aims is to support clinical judgment and help the healthcare team, patients and families exchange on the important topic of QoL. So far, parental and professional versions have been developed. The tool includes a series of instructions, and seven short descriptions, for each domain of QoL. The domains were defined using the words of respondents from the present study and examples were included to optimize understandability. Following recommendations by healthcare professionals and consistent with the observation that a child’s condition can change rapidly in advanced care, we chose a 1-day interval to assess children status [9, 27]. Respondents are asked to complete three simple consecutive tasks: 1) Report their level of agreement about the positive contribution of each QoL domain on the patient’s well-being, 2) provide comments on the reported levels and indicate targets for future interventions, 3) report each level of agreement on a radar chart. In line with the multidimensional QoL model, we kept dimensions independent from one another (no total score is computed). The radar chart offers a graphical overview of the strengths and weaknesses of the patient’s current status as perceived by respondent.

### Content and format validation of Advance QoL tool

Overall, experts positively evaluated the tool’s clarity, relevance, format, and usability. They commented extensively on each section of the tool requiring improvement. Here are the results of the experts’ evaluation of the tool based on the criteria evaluated quantitatively, followed by the adjustments made for each of these criteria according to the qualitative suggestions retained.

### Clarity

Although experts considered instructions as clear (CVI = 0.80), they suggested simplifying the language and offering additional oral explanations to help the respondent follow the steps to complete the tool. Following these recommendations, we clarified the instructions paragraph in the revised version of the tool. We also added a step number (#1, #2, #3) next to each instruction to help respondents navigate the tool.

As for QoL domains, the CVI showed lower levels in the following: Physical (0.60), Psychological (0.47) and Feeling heard (0.73). Experts mentioned that the negative wording used initially to present the physical and psychological domains was confusing. Thus, we rephrased them to harmonize reporting across domains. All domains are now framed positively. For the Feeling heard domain, the original wording invited the respondent to imagine the child’s feelings. This wording was found difficult to understand and experts recommended to ask respondents more directly if they considered that the patients were heard by their entourage and healthcare team. We implemented this accordingly in the revised version.

### Relevance

Data from interviews and the quantitative survey confirmed the tool’s design was coherent with its aim of monitoring patients’ QoL (CVI = 100). Experts considered the tool as a promising instrument to facilitate case management and the selection of appropriate individual targets to improve a patient’s QoL. Experts agreed on the tool’s potential to facilitate communication within the healthcare team (CVI = 93.3) and with the family (CVI = 93.3).

Both in their verbal commentaries and the quantitative survey experts considered the three-point response scale (agree, partially agree, disagree) as relevant (CVI = 100). They also appreciated the opportunity to provide explanations and to identify targets for future action (CVI = 86.7 and 92.9). They reported that this would allow respondents to take a step back and think critically on treatment planning, and perhaps improve decision-making involving the team and the family. One expert stressed that future users of the tool could complete these explanation-targets boxes even when ratings are already high as it could help identify which aspect should be maintained or reinforced.

The radar chart was also rated positively by the experts (CVI = 86.7). They reported it would be adequate to provide a quick overview of the respondent’s perspective of the patient’s QoL and spot domains needing improvement. They also reported it would ease comparisons between raters during case management meetings.

Finally, concerns were reported by the experts about the 1-day timeframe to estimate the patient status (CVI = 0.23). Although experts agreed that the child’s QoL should be assessed over short periods in the present context, they mentioned the difficulty for some professionals to use this timeframe as encounters may be less frequent than once a day (e.g. psychosocial support). Experts suggested that instructions should refer to the last encounter with the patient. Experts also advised us to add an additional box to clarify contextual elements that could influence their current appreciation of QoL. These adjustments were implemented in the last version of the tool.

### Format and usability

The tool was unanimously considered easy to use (CVI = 100). The interviews highlighted the experts’ appreciation of the double-sided page format. To further improve the presentation, experts suggested airing out the description of the dimensions using bullet points. Importantly, they evaluated completion time as adequate for healthcare professionals (CVI = 80) and for parents (CVI = 85.7). These changes were made in the last version. Some experts suggested that in some cases, the family could be supported by a professional during completion in order to provide emotional support.

**Fig. 1:**
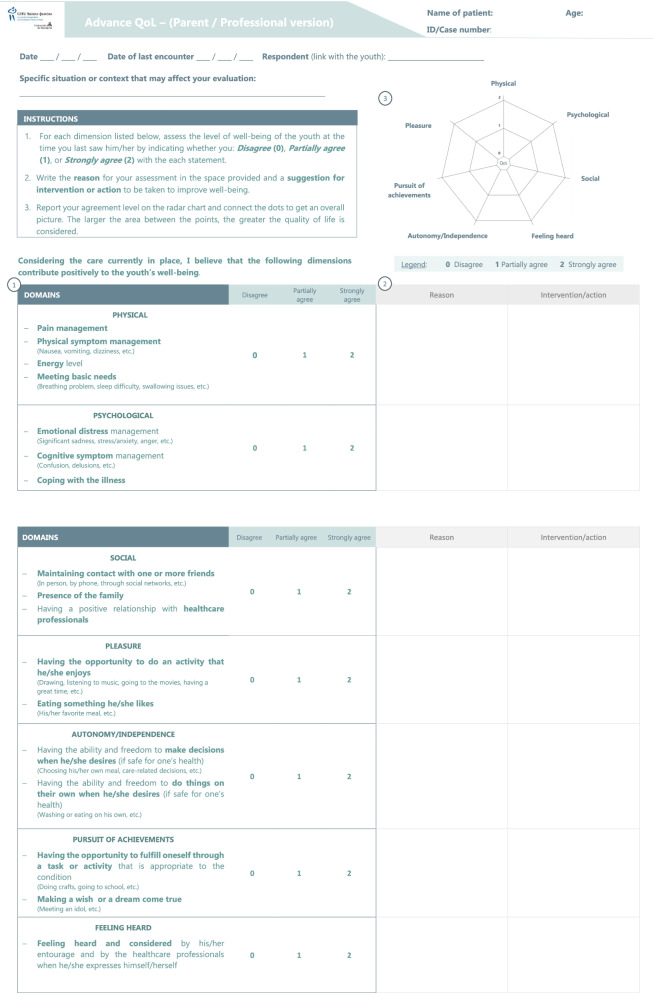
*Advance QoL*: A tool for assessing Quality of Life in children with advanced cancer

To summarize, we collected encouraging quantitative evaluations by experts on the preliminary version of Advance QoL. To improve clarity and relevance, we implemented most of their qualitative suggestions, which led us to a usable refined version (Fig. 1).

## Discussion

In this study, we aimed to identify the domains of QoL from the points of view of patients and parents and to elaborate a simple tool able to grasp the key life domains of this population. In such a context of vulnerability, it was necessary to adopt a careful and rigorous ethical reflection to provide a way of expression to children and parents while offering them optimal protection. As indicated in the method section, several practical measures were adopted. In particular, the person who led the interviews (JAB) had the skills to provide support when needed and her caring approach combined with the collaborative work with clinical teams allowed to ensure flexibility so as not to add to the participants’ burden.

In line with the study purpose, we opted for a qualitative approach. In addition to providing flexibility for adjustment along the course of the study, this approach allowed to extract meaning from the experiences of the children and parents in order to describe in depth the important dimensions of QoL in the context of advanced cancer. Collecting perspectives of children with advanced cancer and their parents by qualitative method was essential to provide information complementary to previous studies based on previously defined theoretical concepts and to validate QoL dimensions previously derived from the perspective of professionals [11]. Knowing how patients and families conceive of QoL in this context made it possible to choose indicators that could reflect key characteristics of QoL domains, an essential preliminary step before developing an assessment tool [8, 15, 16]. Furthermore, we chose to pool different child and parent perspectives, alongside that of the caregivers already available, to provide a more accurate understanding of the concept of QoL in the context of pediatric palliative oncology care. Having multiple sources of information also participate in the phenomenon of triangulation, an aspect that improves the validity of qualitative research.

We found that patients and parents had consistent views, and that they spontaneously identified the same key domains as professionals did in a previous report. The triangulation of the child, parents and professionals’ perspectives made thus possible to formulate a model of QoL for this population. This takes the form of an operational definition based on the following seven domains: physical, psychological, and social well-being, the preservation of autonomy and independence, day-to-day pleasure, the pursuit of achievements, and the sense of feeling heard. We used this definition to guide the design of an evaluation tool, Advance QoL. Consulting with experts, we confirmed its clarity, relevance and usability, and refined it for future use.

Rich descriptions emerged when participants described QoL. As parents highlighted the need for their children to maintain their autonomy and keep connected with previous activities, children insisted on their desire to continue school to see their friends and fulfill themselves. Maintaining autonomy and pursuing achievement, as viewed by both groups, should be adjusted to the clinical context of each patient, their limitations and growth potential. Notably, even in such life-threatening context, participants insisted on fostering growth in the form of offering opportunities to accomplish activities, taking up roles, etc. This result is aligned with recent principles of pediatric palliative care highlighting functioning areas and keeping a positive outlook through a sense of purpose and a certain sense of normalcy [12, 28, 29]. It is also coherent with theories of self-regulation and meaning making, where the sense of achievement and purpose is key to individual well-being in life-threatening circumstances [30, 31]. As did professionals, patients and parents also insisted on the importance of finding sources of pleasure, as it allows patients to refocus on the present moment, offering relief in times of discomfort and limitations due to cancer and treatments [32]. This is reflected in participants’ insistence on wording QoL domains in positive and active terms. The descriptive model emerging from the data provides a positive outlook with most domains presenting true potential for achievement and progress. It is an opportunity to refocus on aspects defining the QoL per se and develop an individual improvement-centered approach. This is in sharp contrast with most existing QoL tools that typically enquire about symptoms or impairments [8, 10]. Importantly, this positive tone focusing on progress was spontaneously offered by families and healthcare professionals. In conclusion, based on an inductive and holistic approach, the results on both the definition of QoL domains and their general positive orientation provided a solid conceptual foundation for developing a specific and individualized tool such as Advance QoL.

When exploring how Advance QoL was perceived, we found an overall high level of satisfaction with the tool. Although some changes were needed to clarify its content and adjust the timeframe, it was perceived as easy to use. Advance QoL meets the need for a brief and pertinent assessment tool of QoL in patients with advanced cancer [1, 5]. Its brevity as well as its radar chart summary provide a quick overview of the respondent’s perception about the patient’s QoL, with minimal burden on the respondent. Importantly, as it is essentially composed of seven one-item scales, completion is kept as simple as possible. These innovative features were well-received by professionals and should further facilitate implementation. As professionals have been involved from the very beginning of the conceptual definition we may expect an excellent uptake in clinical settings [11, 33].

Another important characteristic of Advance QoL noted by experts is its potential to improve teamwork around these complex cases. In the absence of a common QoL model, evaluation and care has remained vulnerable to personal and professional biases that are difficult to reconciliate [11]. This is even more so since in pediatric cancer care is coordinated among increasingly diverse professions. Sharing one QoL model and communicating on agreements and disagreements among respondents may help articulate different views and form a unified portrait of the patient [34]. In addition, consistent with multi-informant assessment strategies, it is paramount to collect reports from both the family and professionals as perceptions may be quite different [35]. These differences may constitute an invaluable source for confirming patients’ status and defining future targets of care [5, 36, 37]. Recent guidelines and priorities have indeed insisted on improving communication and information sharing among clinical teams, patients, and families [1, 38]. Although evaluative studies should be led to prove this, the comments of experts of the present study suggest that Advance QoL could serve this purpose adequately in the future.

We must recognize the limitations of this work. First, although we stopped recruiting when data saturated, the number of participants is still small and the results cannot be generalized. In addition, the fact that some of the participants are from the same family may have had an impact on the recurrence of certain themes. Conducting a multi-center study could offer greater transferability of the study. Studying the pertinence of the tool in a culturally diverse sample should also be performed before implementation. Thus, the present study should be considered as a preliminary refining step of a simple instrument applied to a French Canadian context. Hence, future studies should focus on exploring its reliability, validity, and assess the feasibility of the implementation of the tool in the clinical setting. Second, only one version has been developed so far (professional and parent version). Finally, as it is essential to collect the perception of patients, future studies should develop versions appropriate for children and adolescents.

Despite these limitations, the study offers a rigorous conceptual and methodological foundation [39, 40]. By collecting complementary viewpoints on QoL from children and parents, and adding to existing previous work with healthcare professionals, we triangulated data to refine and enrich the QoL model. We ensured the scientific rigour of the QoL model conceptualization phase by using a reflexive journal and called upon intercoder agreement. As a second step, we tested the content and form of the tool by collecting a structured feedback from expert end-users. The integration of this feedback in the refinement of Advance QoL may be considered as a respondent validation strengthening credibility of the research [39].

## Conclusion

In this qualitative study on the definition of QoL in the context of advanced cancer, we found that views of patients and parents were consistent. Considering these views with those of professionals collected previously, we established a model of QoL based on seven domains: physical, psychological, social, autonomy/independence, pleasure, pursuit of achievements, and feeling heard. We developed Advance QoL, an innovative multidimentionnal and individualized schedule to assess QoL and improve communication among clinical teams, patients, and families. We tested it in a pool of experts to collect structured feedback. Quantitative and qualitative results confirmed its clarity, relevance, format adequacy, and usability. Future work should adapt Advance QoL for comprehension levels of children and adolescents, study its validity and its implementation in practice to better serve this vulnerable population.

## Supplementary Information

**Additional file 1.**

**Additional file 2.**

## Data Availability

Data sharing is not possible for this study for ethical reasons as individual privacy will be compromised.

## References

[1] Weaver MS, Heinze KE, Kelly KP, Wiener L, Casey RL, Bell CJ (2015). Palliative care as a standard of care in pediatric oncology. Pediatr Blood Cancer.

[2] Snaman J, McCarthy S, Wiener L, Wolfe J (2020). Pediatric palliative care in oncology. J Clin Oncol.

[3] Schenker Y, Arnold R (2017). Toward palliative care for all patients with advanced cancer. JAMA Oncol.

[4] Ameringer S, Macpherson CF, Jibb L. Symptom science in pediatric oncology. Pediatr Oncol Nurs. Cham: Springer; 2020. p. 79–93.

[5] Tomlinson D, Yuan C, Cheng L, Hinds PS, Hinds P, Linder L (2020). Patient-reported outcomes in pediatric oncology: the voice of the child. Pediatric oncology nursing.

[6] Menard JC, Hinds PS, Jacobs SS, Cranston K, Wang J, DeWalt DA (2014). Feasibility and acceptability of the patient-reported outcomes measurement information system measures in children and adolescents in active cancer treatment and survivorship. Cancer Nurs.

[7] Schepers SA, Sint Nicolaas SM, Haverman L, Wensing M, Schouten van Meeteren AY, Veening MA (2017). Real-world implementation of electronic patient-reported outcomes in outpatient pediatric cancer care. Psychooncology.

[8] Friedel M, Aujoulat I, Dubois A-C, Degryse J-M (2019). Instruments to measure outcomes in pediatric palliative care: a systematic review. Pediatrics.

[9] Coombes LH, Wiseman T, Lucas G, Sangha A, Murtagh FE (2016). Health-related quality-of-life outcome measures in paediatric palliative care: a systematic review of psychometric properties and feasibility of use. Palliat Med.

[10] Huang I-C, Shenkman EA, Madden VL, Vadaparampil S, Quinn G, Knapp CA (2010). Measuring quality of life in pediatric palliative care: challenges and potential solutions. Palliat Med.

[11] Avoine-Blondin J, Parent V, Fasse L, Lopez C, Humbert N, Duval M (2018). How do professionals assess the quality of life of children with advanced cancer receiving palliative care, and what are their recommendations for improvement?. BMC Palliat Care.

[12] Cataudella D, Morley TE, Nesin A, Fernandez CV, Johnston DL, Sung L (2014). Development of a quality of life instrument for children with advanced cancer: the pediatric advanced care quality of life scale (PAC-QoL). Pediatr Blood Cancer.

[13] Morley TE, Cataudella D, Fernandez CV, Sung L, Johnston DL, Nesin A (2014). Development of the pediatric advanced care quality of life scale (PAC-QoL): evaluating comprehension of items and response options. Pediatr Blood Cancer.

[14] Avoine-Blondin J, Parent V, Lahaye M, Humbert N, Duval M, Sultan S (2017). Identifying domains of quality of life in children with cancer undergoing palliative care: a qualitative study with professionals. Palliat Support Care.

[15] Streiner DL, Norman GR, Cairney J (2015). Health measurement scales: a practical guide to their development and use.

[16] Turner RR, Quittner AL, Parasuraman BM, Kallich JD, Cleeland CS, Group MFP-ROCM (2007). Patient-reported outcomes: instrument development and selection issues. Value Health.

[17] Guest G, Namey E, Chen M (2020). A simple method to assess and report thematic saturation in qualitative research. Plos One.

[18] Fusch PI, Ness LR (2015). Are we there yet? Data saturation in qualitative research. Qual Rep.

[19] Saunders B, Sim J, Kingstone T, Baker S, Waterfield J, Bartlam B (2018). Saturation in qualitative research: exploring its conceptualization and operationalization. Qual Quant.

[20] Braun V, Clarke V (2006). Using thematic analysis in psychology. Qual Res Psychol.

[21] Paillé P, Mucchielli A (2012). L'analyse qualitative en sciences humaines et sociales.

[22] Hinds PS, Gattuso JS, Fletcher A, Baker E, Coleman B, Jackson T (2004). Quality of life as conveyed by pediatric patients with cancer. Qual Life Res.

[23] Fonteyn ME, Kuipers B, Grobe SJ (1993). A description of think aloud method and protocol analysis. Qual Health Res.

[24] Van Someren M, Barnard Y, Sandberg J (1994). The think aloud method: a practical approach to modelling cognitive.

[25] Lynn MR. Determination and quantification of content validity. Nurs Res. 1986;35(6):382-5. 3640358

[26] Yusoff MSB. ABC of content validation and content validity index calculation. Resource. 2019;11(2):49-54.

[27] Watanabe SM, Nekolaichuk C, Beaumont C, Johnson L, Myers J, Strasser F (2011). A multicenter study comparing two numerical versions of the Edmonton symptom assessment system in palliative care patients. J Pain Symptom Manag.

[28] Barrera M, D'Agostino N, Gammon J, Spencer L, Baruchel S (2005). Health-related quality of life and enrollment in phase 1 trials in children with incurable cancer. Palliat Support Care.

[29] Friedrichsdorf SJ, Postier A, Dreyfus J, Osenga K, Sencer S, Wolfe J (2015). Improved quality of life at end of life related to home-based palliative care in children with cancer. J Palliat Med.

[30] Scheier MF, Carver CS, Cameron LD, Leventhal H (2003). Goals and confidence as self-regulatory elements underlying health and illness behavior. The self-regulation of health and illness behaviour.

[31] La Cour K, Johannessen H, Josephsson S (2009). Activity and meaning making in the everyday lives of people with advanced cancer. Palliat Support Care.

[32] Friedrichsdorf SJ, Zeltzer L, Kreitler S, Ben-Arush M, Martin A (2012). Palliative care for children with advanced cancer. Pediatric psycho-oncology: psychosocial aspects and clinical interventions.

[33] Langer L, Tripney J, Gough DA (2016). The science of using science: researching the use of research evidence in decision-making.

[34] Feraco AM, Brand SR, Mack JW, Kesselheim JC, Block SD, Wolfe J (2016). Communication skills training in pediatric oncology: moving beyond role modeling. Pediatr Blood Cancer.

[35] Abate C, Lippé S, Bertout L, Drouin S, Krajinovic M, Rondeau É (2018). Could we use parent report as a valid proxy of child report on anxiety, depression, and distress? A systematic investigation of father–mother–child triads in children successfully treated for leukemia. Pediatr Blood Cancer.

[36] De Los RA, Augenstein TM, Wang M, Thomas SA, Drabick DA, Burgers DE (2015). The validity of the multi-informant approach to assessing child and adolescent mental health. Psychol Bull.

[37] De Los RA, Kazdin AE (2005). Informant discrepancies in the assessment of childhood psychopathology: a critical review, theoretical framework, and recommendations for further study. Psychol Bull.

[38] Baker JN, Levine DR, Hinds PS, Weaver MS, Cunningham MJ, Johnson L (2015). Research priorities in pediatric palliative care. J Pediatr.

[39] Mays N, Pope C (2000). Assessing quality in qualitative research. BMJ.

[40] Whittemore R, Chase SK, Mandle CL (2001). Validity in qualitative research. Qual Health Res.

