# Supplementary material for: Quality of life in childhood advanced cancer: from conceptualization to assessment with the Advance QoL tool
Source: BMC Palliat Care. 2022 Aug 1;21:138. doi: 10.1186/s12904-022-01025-z (PMC9341040; doi:10.1186/s12904-022-01025-z)
Supplement: Supplementary file 2 — Additional file 2. [file 12904_2022_1025_MOESM2_ESM.docx]

**Supplementary material A**

Interview schedule used with patients and parents in the context of advanced childhood cancer (adapted from Hinds et al., 2004, Quality of Life Research, 13(4), 761-772)

**Interview guide (children)**

**First part of the interview**

1. Could you tell me what is a good day for you?
   - Do you have an example?
2. Could you tell me what is a bad day for you?
   - Do you have an example?
3. What is the most important for you to have a good day?
   - Do you have an example?

**Second part of the interview**

1. In an earlier stage of the research, I met 20 healthcare professionals and asked them to tell me what they thought would be the most important aspects to ensure that children they care for, like you, would live the best possible day. So, I'm going to show you, one at a time, cards with these elements written on them and ask you to tell me if, this element is important or not to live the best possible day. If it's important, please put the card here on the right, otherwise put it here on the left, OK?

**Third part of the interview: domain formulation**

1. Now, when you look at these 7 cards representing the broad domains to have a good day, do you think there are things that should be changed or added to make sure you have the best possible day?

Is there anything else you would like to add about this interview?

**Interview guide (parents)**

**First part of the interview**

1. Could you tell me what is a good day for your child?
   - Do you have an example?
2. Could you tell me what is a bad day for your child?
   - Do you have an example?
3. What is the most important for your child to have a good day?
   - Do you have an example?

**Second part of the interview**

1. In an earlier stage of the research, I met 20 healthcare professionals and asked them to tell me what they thought would be the most important aspects to ensure that children they care for, like your child, would live the best possible day. So, I'm going to show you, one at a time, cards with these elements written on them and ask you to tell me if this element is important or not for your child to live the best possible day. If it's important, please put the card here on the right, otherwise put it here on the left, OK?

**Third part of the interview: domain formulation**

1. Now, when you look at these 7 cards representing the broad domains to have a good day, do you think there are things that should be changed or added to make sure your child has the best possible day?

Is there anything else you would like to add about this interview?

**Supplementary material B**

Assessment grid used by experts to evaluate the clarity, relevance, format, and usability of the Advance QoL tool

**QUALITY OF LIFE TOOL EVALUATION GRID**

The purpose of this grid is to evaluate the quality of the tool developed to assess Quality of Life of children with advanced cancer. Your evaluation will be considered to perform a preliminary validation of this tool.

Referring to the tool, please assess the clarity of the following elements:

**Clarity of content**

|  | **Unclear** | **Clear** | **Remarks** |
| --- | --- | --- | --- |
| **Instructions** paragraph | **1** | **2** |  |
| Description **Physical** domain | **1** | **2** |  |
| Description **Psychological** domain | **1** | **2** |  |
| Description **Social** domain | **1** | **2** |  |
| Description **Pleasure** domain | **1** | **2** |  |
| Description **Autonomy** domain | **1** | **2** |  |
| Description **Pursuit of achievements** domain | **1** | **2** |  |
| Description **Feeling heard** domain | **1** | **2** |  |

**Assessment of the general characteristics of the tool**

| Using the tool, rate the following elements (“1” means strongly disagree and “5” means strongly agree). | **Level of agreement** | | | | | **Remarks** |
| --- | --- | --- | --- | --- | --- | --- |
| **The rating scale is adequate.** | **1** | **2** | **3** | **4** | **5** |  |
| **The « Reasons » section is useful to understand the rating and articulate with targets.** | **1** | **2** | **3** | **4** | **5** |  |
| **The « Target » section is useful to identify interventions opportunities in each of the domains.** | **1** | **2** | **3** | **4** | **5** |  |
| **The overview provided by the Radar chart makes it possible to quickly identify priority targets for action.** | **1** | **2** | **3** | **4** | **5** |  |
| **The completion time of the tool is adequate for healthcare professionals.** | **1** | **2** | **3** | **4** | **5** |  |
| **The completion time of the tool is adequate for parents.** | **1** | **2** | **3** | **4** | **5** |  |
| **The tool is useful for fostering communication within the team about the patient’s quality of life.** | **1** | **2** | **3** | **4** | **5** |  |
| **The tool is useful for fostering communication with parents about the patient’s quality of life.** | **1** | **2** | **3** | **4** | **5** |  |
| **The time reference period (last 24 hours) to assess the youth’s quality of life is appropriate.** | **1** | **2** | **3** | **4** | **5** |  |
| **The tool is easy to use.** | **1** | **2** | **3** | **4** | **5** |  |
| **The tool is useful to help health professionals maintain or improve the youth’s quality of life.** | **1** | **2** | **3** | **4** | **5** |  |

Please insert your additional comments below.

Thank you for taking the time to evaluate this first version of the Advance QoL tool!

**Supplementary material C**

Advance QoL: A tool for assessing Quality of Life in children with advanced cancer (French-language version)

**
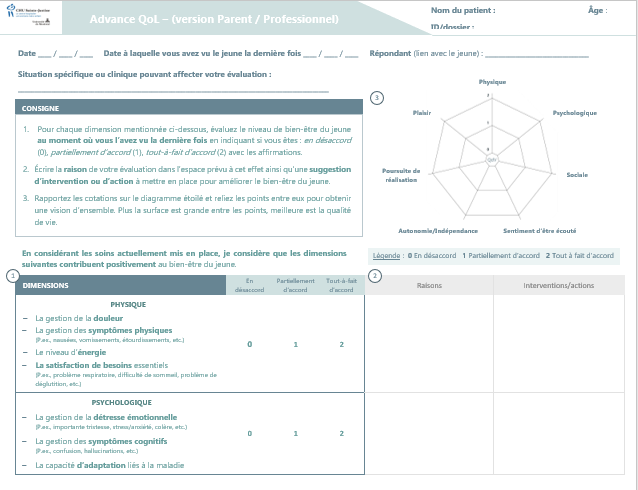
**

**
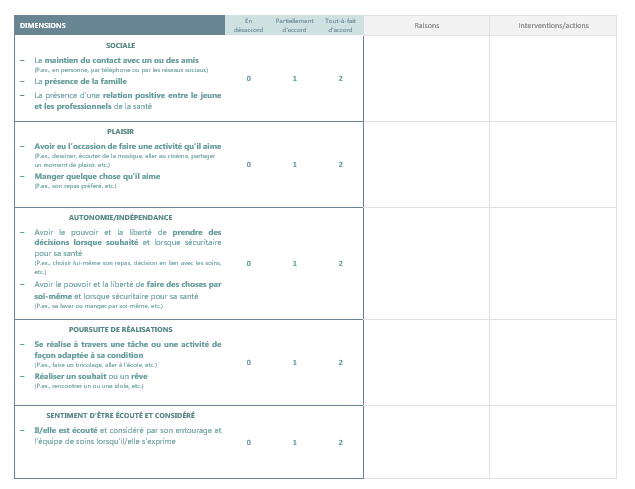
**
